# A Large Area Tactile Sensor Patch Based on Commercial Force Sensors

**DOI:** 10.3390/s110505489

**Published:** 2011-05-19

**Authors:** Fernando Vidal-Verdú, Maria Jose Barquero, Julián Castellanos-Ramos, Rafael Navas-González, Jose Antonio Sánchez, Javier Serón, Alfonso García-Cerezo

**Affiliations:** 1 Department of Electronics, University of Málaga, Málaga 29071, Spain; E-Mails: mjbarquero@altracorporacion.es (M.J.B.); jcramos@uma.es (J.C.-R.); rjnavas@uma.es (R.N.-G.); jsd@uma.es (J.A.S.); 2 Departamento de Ingeniería de Sistemas y Automática, University of Málaga, Málaga 29071, Spain; E-Mails: jseron@uma.es (J.S.); Alfonso.garcia@isa.uma.es (A.G.-C.)

**Keywords:** tactile sensors, assistive robots, human-machine interaction, force sensing resistors

## Abstract

This paper reports the design of a tactile sensor patch to cover large areas of robots and machines that interact with human beings. Many devices have been proposed to meet such a demand. These realizations are mostly custom-built or developed in the lab. The sensor of this paper is implemented with commercial force sensors. This has the benefit of a more foreseeable response of the sensor if its behavior is understood as the aggregation of readings from all the individual force sensors in the array. A few reported large area tactile sensors are also based on commercial sensors. However, the one in this paper is the first of this kind based on the use of polymeric commercial force sensing resistors (FSR) as unit elements of the array or *tactels*, which results in a robust sensor. The paper discusses design issues related to some necessary modifications of the force sensor, its assembly in an array, and the signal conditioning. The patch has 16 × 9 force sensors mounted on a flexible printed circuit board with a spatial resolution of 18.5 mm. The force range of a tactel is 6 N and its sensitivity is 0.6 V/N. The array is read at a rate of 78 frames per second. Finally, two simple application examples are also carried out with the sensor mounted on the forearm of a rescue robot that communicates with the sensor through a CAN bus.

## 1. Introduction

Tactile sensors are basically arrays of force sensors that enable a whole specific surface area to be monitored, instead of only discrete point pressure monitoring. They are demanded in applications where unstructured environments or uncertainty are present, such as minimally invasive surgery (MIS), robotics, rehabilitation, virtual reality, telepresence, or industrial automation [1]. Many different approaches have been proposed to manufacture these sensors, most of them are based on piezoresistive or capacitive principles, and a few are based on optical or piezoelectrical transduction [2]. Many of these sensors are made with silicon or polymers using microelectromechanical systems (MEMS) technologies. These technologies are not oriented to large area devices, so they are usually proposed for applications that demand high spatial resolution and good performance in terms of errors, such as MIS. However, large area devices can be made with skin patches, *i.e*., by connecting several arrays in more complex structures. For instance, a piezoresistive MEMS on polymer sensor that covers an area of 25 mm × 25 mm is presented in [3]. The scalability to larger sensors is limited by the wiring complexity and the authors proposed the addition of silicon based integrated circuits on the same substrate to achieve a modular and large area sensor. Another modular approach based on capacitive MEMS on polymer is examined in [4]. Other implementations build a sensor with MEMS on silicon and soldered in an array on a flexible PCB [5]. Nevertheless, those sensors that are not oriented to cover the fingertips but for example the forearms, do not require a high spatial resolution and are usually developed with other technologies.

Optical tactile sensors are composed of photo emitter and photo detector pairs. Pressure against the sensor modulates the light that is captured by the detector. A skin that covers the whole surface of a robot and allows a safe interaction with humans is presented in [6]. It is made with infrared LED and detector pairs that implement proximity sensors. In [7] a module is presented with 32 tactels that are based on a LED, a phototransistor and a urethane foam atop. The amount of light scattered in the foam and detected by the phototransistor depends on the pressure exerted on the tactel. Quite high hysteresis is observed. In [8] sensors based on fiber Bragg gratings are proposed. The pressure causes a shift in the wavelength of the Bragg grating. The tactels have good sensitivity, repeatability and no hysteresis. However, each tactel has its own Bragg wavelength, which is a drawback to building large arrays (the paper shows results from small arrays of 3 × 3 elements), and the electronics are complex.

Capacitive tactile sensors exploit the dependence of the capacitance on the distance between the plates of a capacitor that has a deformable dielectric layer. Parasitic capacitors and noise are major concerns, so signal conditioning must be close to the raw sensor. Conformable and stretchable capacitive tactile sensors are commercialized by Pressure Profile Systems [9]. In [10] a modular approach is proposed where triangular modules of 12 capacitive tactels are connected to each other to cover any shape. An off-the-shelf CDC capacitive to digital converter (AD7147) standard chip from Analog Devices is used for signal conditioning in every module. A common drawback of tactile capacitive sensors is hysteresis. However in [11] a sensor is presented that has no hysteresis. This is achieved by an appropriate selection of the elastomer between capacitor plates. Signal conditioning is carried out with multivibrators made with off-the shelf integrated circuits.

The last and larger group of sensors reported to cover large areas are those based on piezoresistive principles. Several are made of conductive fabric or rubbers [12,13]. A common method of producing these sensors consists in implementing an array of electrodes on a flexible printed circuit board, and a conductive rubber or polymer is placed atop of them [14,15]. QTC (Quantum Tunneling Composite) from Peratech is used in [16] to build the array (64 tactels in the forearm) of custom sized sensors cut from A4 size sheets. Wiring is again a concern here. The EIT (Electrical Impedance Tomography) technique is used in [17] to implement a stretchable tactile sensor that has electrodes only at the contour, thus wiring is reduced. However, tactile sensors based on EIT are less accurate and the procedure to read the data from them is quite slow. A sensor is proposed in [18] that communicates through microwaves in a two dimensional sheet. It implements a RFID tag and a resonant proximity connector in every tactel and the whole array detects binary images (it does not register pressure maps). The sensor in [19] also provides a binary output and points to a printing technology plus multiplexing to reduce the number of external wires. The sensor in [20] is also made with a large-area printing technology and addressing is done with a switching matrix of organic field-effect transistors implemented on the same substrate. This is a promising technology, although the estimated scan time in a 16 × 16 array is 480 ms.

Other authors take advantage of the low spatial resolution of the tactile sensor and relatively large size of the tactel to implement it with a commercial force sensor. In [21] this approach is adopted with silicon based force sensors. These sensors have good performance in terms of drift, hysteresis or linearity. However, pieces of elastic material are used to concentrate the force at the diaphragms of the pressure-sensing elements and another elastic sheet is used to make the sensor soft and perform a spatial filtering. The addition of these elastic materials degrades somewhat the performance. Moreover, silicon is fragile and brittle in comparison to other materials like polymers. In [22] polymeric FSRs (Force Sensing Resistors) are used as on-off sensors in combination with force-torque sensors to compute the magnitude and direction of the force and the contact position. However, it is assumed that a simple force is applied, so its application is limited when more complex forces are exerted and more detailed information is required, as in the case of holding a human being in the arms of the robot. Similar sensors have been used to provide some tactile sensitivity to robotic hands in [23], where they are not used as binary sensors but the whole output force range is exploited. Only a few of these sensors are necessary in [23] and they are wisely located in the robotics hands.

This paper presents a large area tactile sensor that is made of the same commercial FSRs soldered on a flexible Printed Circuit Board (PCB). Some preliminary results were presented in [24]. Many problems arise when these FSRs are intended to be used in this way. Some modifications have to be made to the sensor to preserve its performance and behavior as an isolated element. The paper explains these modifications and the strategies followed to build the patch and minimize errors and interferences. The obtained tactel is characterized and results for the input-output curve, drift, step response and mismatching are shown in the paper. The force range of the tactel is 6 N and its sensitivity is 0.6 V/N. The patch has 16 × 9 tactels with a spatial resolution of 18.5 mm and it is read at a rate of 78 frames per second. Signal conditioning is based on a PIC18F4680. A modular approach is achieved in this way since communication with a CAN bus is also implemented, so many of these or similar patches can be connected. Two application examples were also carried out where the sensor was used to cover the forearm of the ALACRANE rescue robot [25]. Details are given at the end of the paper.

### 2. Specifications of the Sensor

As a reference to design the sensor, we are interested in avoiding violent collisions that can hurt humans. When a human being comes into contact with the surface of an object the amount of force produced is typically around 0.1–2.0 N and this force can be taken as a reference for the minimum value to be detected and does not cause any pain. Regarding maximum force, the average pressure exerted against the skin by the weight of a body held in the arms is around 14 kPa (0.15 Kg/cm^2^ aprox.) and a certain security margin must be taken [21]. The response time in which a human completes all processing of tactile stimuli from contact detection to response output is 100–200 ms. This is taken in [22] as a reference of the input-output delay of the smart tactile sensor. As regards to the spatial resolution, the static simultaneous two-point discrimination threshold of the human skin in the forearm is around 38 mm [26]. In the following section we will describe the design of a sensor that meets these specifications. Design issues related to the raw sensor and electronics are discussed.

## 3. Design of the Raw Sensor

As mentioned in the introduction, our design is based on commercial force sensing resistors (FSR). Three commercial FSRs from Lusense, Tekscan (Flexiforce sensors) and Interlink Electronics are on the market. These sensors have been compared in [27] and [28]. The FSRs from Interlink [29] are the most robust, and this is an important point in our design because of the hostile environment and heavy weights which the robot has to cope with. Moreover, these sensors have a good tradeoff between drift, hysteresis and accuracy. A few different sensors are available from Interlink Electronics, two round in shape, one square and another strip shaped one. Round sensors are more suitable for forming our array. The larger one was chosen (Interlink Electronics Standard 402 FSR) [29] because it allows the resolution requirements to be accomplished of less than 38 mm between tactels. In addition it also fits our force requirements. Table 1 lists the main performance data of this sensor.

**Figure 1.**
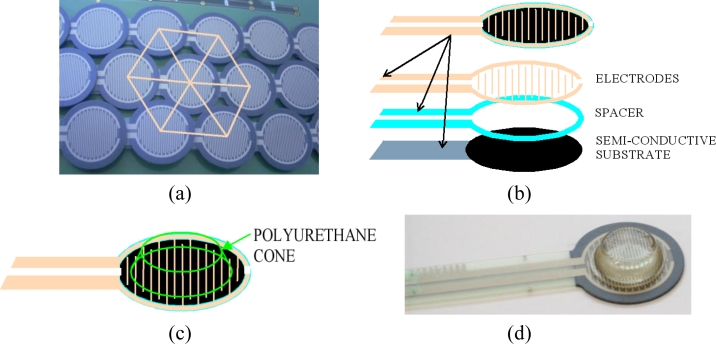
(**a**) Detail of the grid. (**b**) Layers in a Force Sensing Resistors from Interlink. (**c**) Draw of the sensor with a polyurethane cone atop. (**d**) A photograph.

We had to decide how to arrange the sensors in a flexible sheet that is shaped as a cylinder when placed in its final destination. In order to have the best filling factor, we decided to arrange them in a triangular grid (see Figure 1(a)).

A slight, but significant, modification of the sensor refers to the spacer the commercial sensor has between the layer with the electrodes and the flexible substrate with printed semi-conductor (see Figure 1(b)). This spacer guarantees that there is no response in absence of force applied to the sensor. However, the presence of this spacer causes no response even in the case that a flat rigid object presses the sensor, because it avoids the contact between the substrate and the electrodes. This is observed clearly in our sensor. It does not register any data with the above described array of FSRs. Some element must be added atop to allow the force to reach the active area of the sensor, which is the inner area beyond the spacer. A possible solution we have adopted is the addition of small polyurethane cones (circular bumpers BS-01R, Durometer, Shore A 60–70 Standard [30]) atop each FSR, as Figure 1(c,d) illustrate. Trials with continuous sheets of deformable materials were also made. The conclusion was that the response depends greatly on the properties of the material. We registered readings with quite flexible foams but not with other more rigid elastomers atop the sensor. With a single piece of elastomer per sensing resistor, any pressure exerted against a tactel is translated by the cone directly to the active area of the FSR. Moreover, this approach allows the characterization as an isolated element.

**Figure 2.**
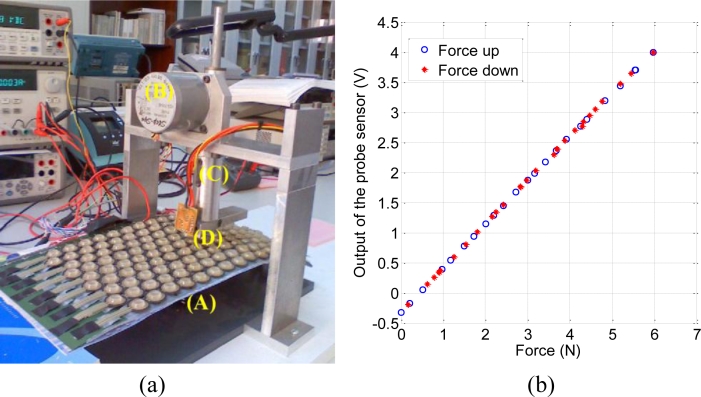
(**a**) Characterization set-up. (**b**) Calibration curve.

The influence of the added cone on the performance of the sensor was tested with some experiments. We used the characterization set-up of Figure 2(a). Basically, it consists of a translation stage (A) to place the sensor on, a stepper motor (B) to exert the force via a spring (C) and finally a force sensor placed at the tip of the probe (D) (Honeywell FSG15N1A). The measurement process is automatic and it is controlled by a computer. The computer sends the commands to the motor and reads the data from the sensor by means of a data acquisition board. The system is calibrated before making the measurements. It is done by pressing with the probe against a precision balance (KERN 440-47N) and taking the readings from the balance and from the characterization set-up to obtain the calibration curve in Figure 2(b).

**Figure 3.**
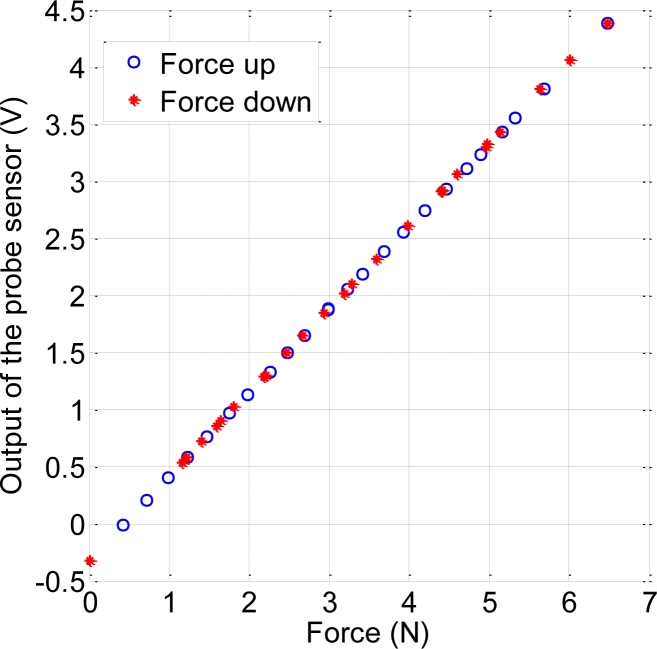
Data obtained when the probe presses against a polyurethane cone placed on the balance.

A first simple test to know the influence of the polyurethane cone was carried out. It consisted of repeating the calibration procedure but placing a cone between the probe and the balance. The curve obtained is shown in Figure 3. The larger the difference between this curve and the calibration curve in Figure 2(b), the larger the influence of the cone would be. Since this difference is negligible, we can conclude that the piece of polyurethane does not introduce a significant disturbance in terms of hysteresis or linearity.

**Figure 4.**
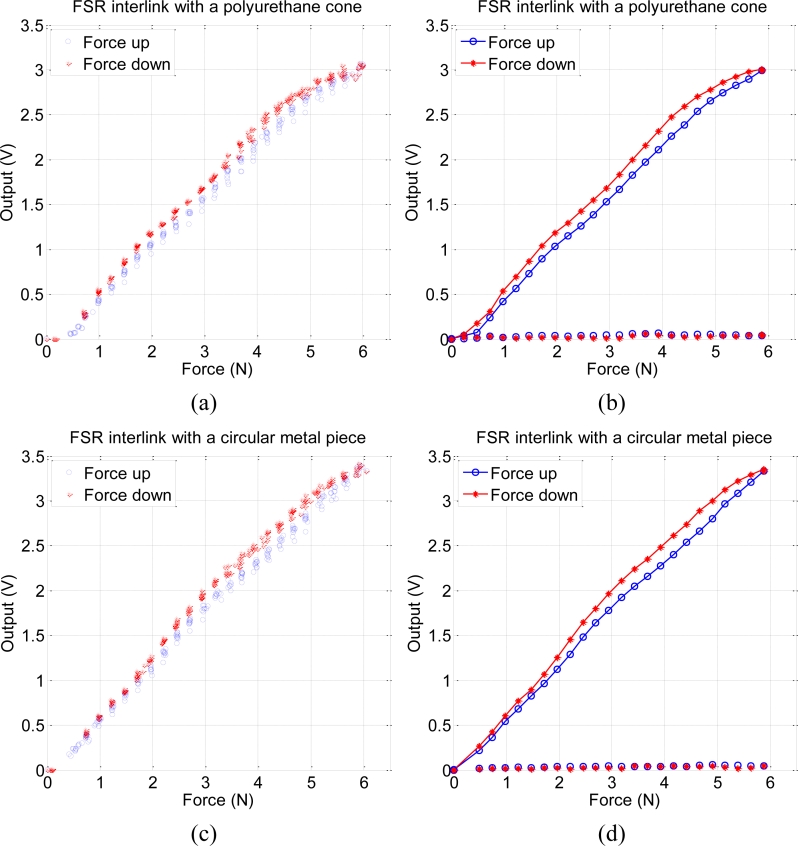
(**a** and **b**) Static response to a pressure up—pressure down cycle of the modified sensor with the cone atop of one sensing resistor. (**c** and **d**) Static response of the sensor without the polyurethane cone and pressed with a circular piece of metal and a small piece of fabric at the interface with the sensor to achieve a uniform contact pressure.

Another test was done with the cone atop of one sensing resistor, and both attached on a rigid and flat surface. The data registered by the set-up in Figure 2(a) is shown in Figure 4(a,b). On the left, the readings from ten cycles of increasing and decreasing force are shown while the figure on the right depicts the mean value and standard deviation obtained from this data. The same measurement was made but replacing the polyurethane cone with a circular piece of metal and a small piece of fabric at the interface with the sensor to achieve a uniform contact pressure. The results are in Figure 4(c,d), where the meaning of data is the same as in Figure 4(a,b). The curves are also very similar this time, with no significant differences in terms of errors. The gain is slightly larger in the case of the circular piece, but it is because its diameter is 11.5 mm while the diameter of the basis of the cone is 10.2 mm. The diameter of the active area of the sensing resistor is 12.7 mm, so a cone that fitted this area would perform better.

**Figure 5.**
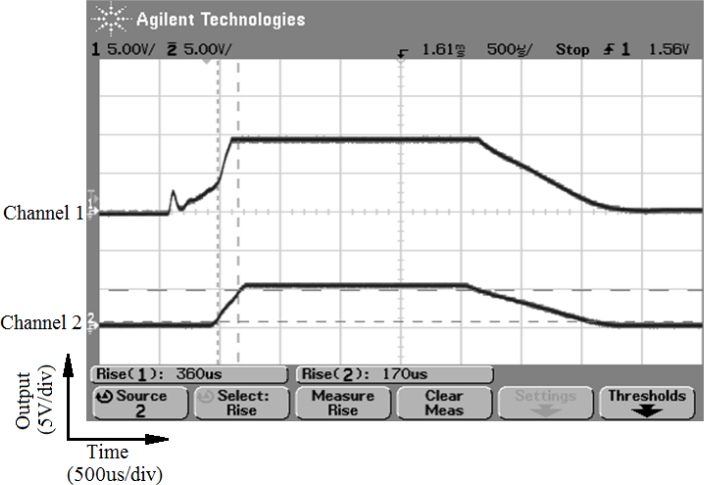
Tactel response to a force pulse. Channel 1 shows the output from the probe silicon force sensor and Cannel 2 shows the output from the tactel.

Two additional tests were made to establish the dynamic response of the modified sensor. FSRs have a rise time between 1–2 ms determined by their mechanical design. A simple test was made to assure the modification with the polyurethane cone did not change this dynamic response. Figure 5 shows the response to a force pulse measured by the silicon force sensor (top curve) and by the modified FSR (bottom curve). Responses are in the order of hundreds of microseconds, so they do not determine the limitation of the dynamic response of the sensor to meet a delay below 200 ms. This limitation will be given by the electronics discussed in the next section.

**Figure 6.**
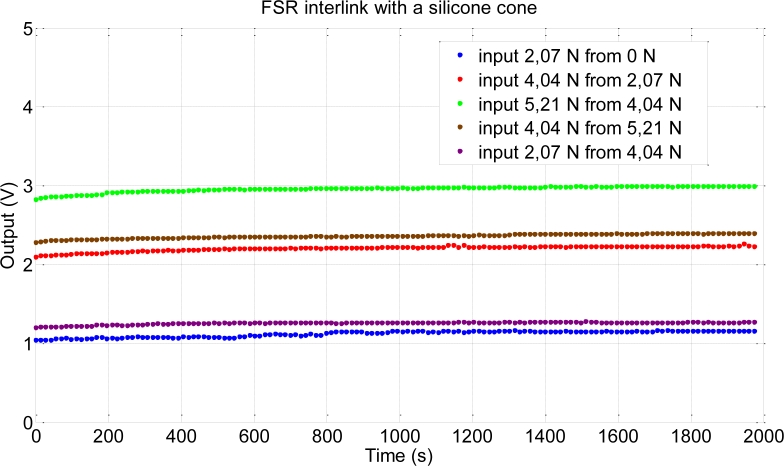
Measured drift of a tactel.

Regarding drift, we have followed a procedure similar to that reported in [28] for a characterization of commercial force sensors based on similar principles. This procedure consists in measuring the drift not only when pressure is exerted on the sensor from a prior situation of no pressure on it, but also the result of a few increments from starting pressure different to zero. We also show results of positive as well as negative increments. Figure 6 shows the results of this experiment that was performed with the set-up in Figure 2(a). A drift of up to 10.7% of the full scale output in 1,974 s is observed in the worst case. The largest drift is observed when the starting pressure is zero. This effect could be reduced by applying a preload.

**Figure 7.**
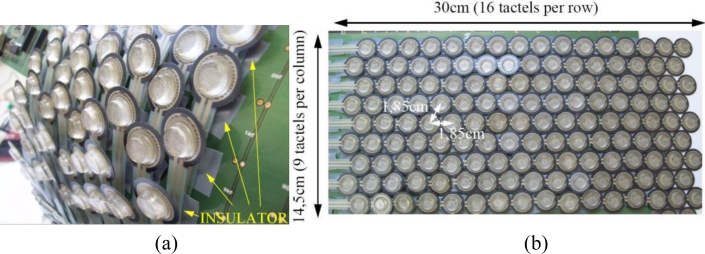
(**a**) Detail of the placement of the sensors, and (**b**) a photograph of the whole array.

The FSR sensors from Interlink also have long leads with the connectors at their ends. These connectors are crimped because the leads are damaged by the heat and cannot be soldered directly to the PCB. As a consequence, the distance between FSRs in the array is very large unless we allow they overlap. We could cut the leads and crimp them again, but this would take a long time. Moreover, it is not possible to remove them completely and the distance between tactels would be always larger than if the force sensors overlap. Therefore, active areas of the sensors lie on the leads of others (see Figure 7) and the spatial resolution is optimum.

**Figure 8.**
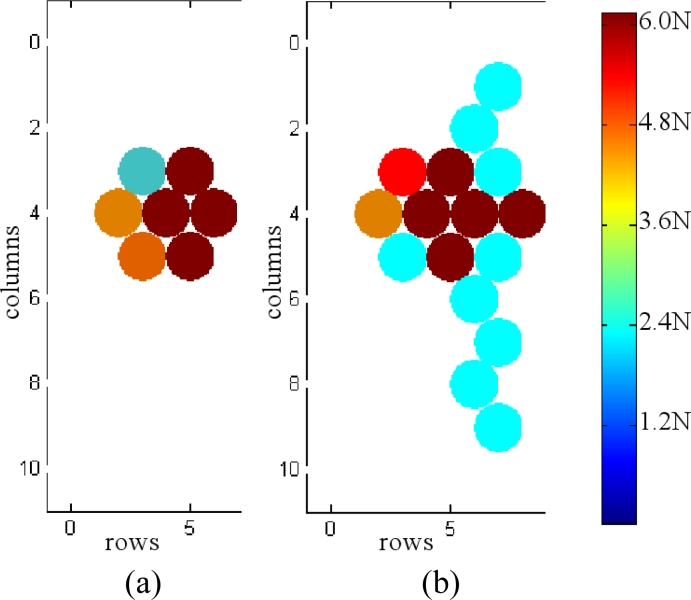
Tactile image of a circular object without (**a**) and with (**b**) insulating the solderings.

Two more practical issues are important to discuss here. First, we observed that interferences appear despite the electronic (described in the next section) being designed to cancel them. After investigations we saw the contact of the sensor leads with solderings of other tactels caused such interferences. Therefore, insulation of the solderings was required to remove them, as Figure 7(a) shows. Figure 8 shows the reading of the sensor when it is pressed with a circular piece of metal with and without insulating the solderings.

**Figure 9.**
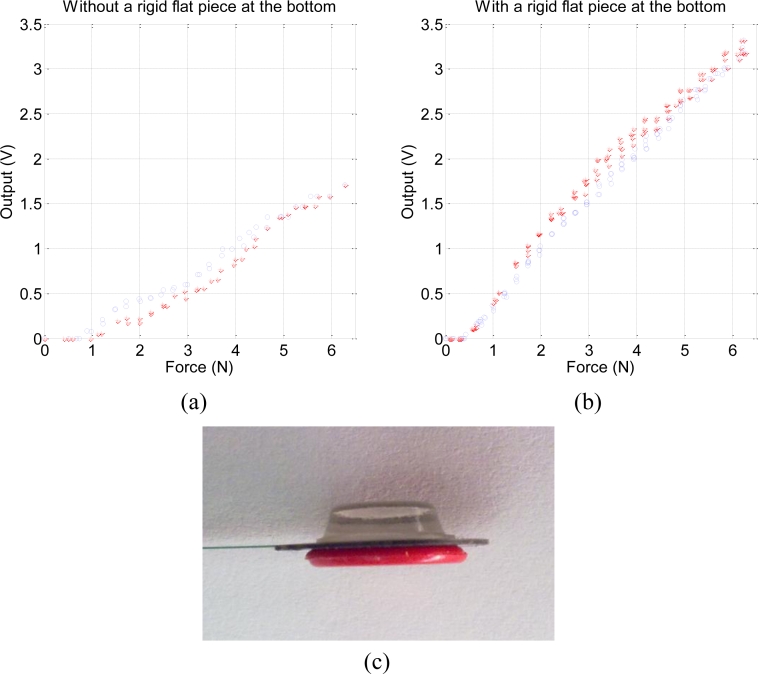
(**a**) Tactel output without and (**b**) with a rigid flat piece at the bottom. (**c**) Photograph of the modified FSR.

The other implementation issue refers to the convenience of having a flat and firm surface under the force sensing resistor. If the sensors are mounted as Figure 7 depicts and they rest on the tails of other sensors, a poor performance is observed. For instance, Figure 9(a) shows the output obtained with the characterization set-up of Figure 2(a) when the output from one of the mounted tactels is registered. A highly non-linear and distorted output is obtained. Another modification of the sensor is required to achieve an output similar to that reported by the manufacturer. It is done by attaching a circular rigid plastic piece at the bottom of the sensor, as Figure 9(c) shows. In this way the output from the sensor is quite linear, as Figure 9(b) shows.

Finally, another test was carried out to obtain information about the mismatching of the sensors in the array, as in [21]. The output of 16 tactels was read with the set-up in Figure 2(a) and the results are shown in Table 2. The variations could be tolerated for practical purposes [1], or some calibration procedure can be implemented that takes advantage of the microcontroller of the sensor.

## 4. Design of the Electronics

**Figure 10.**
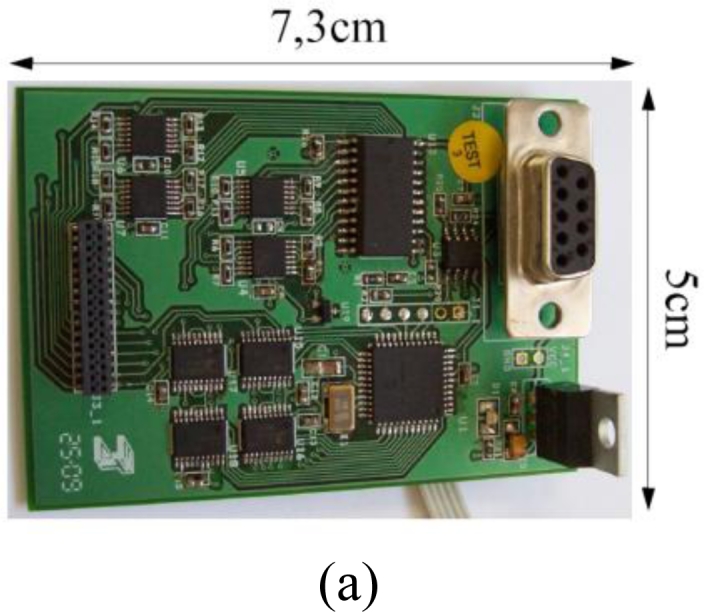

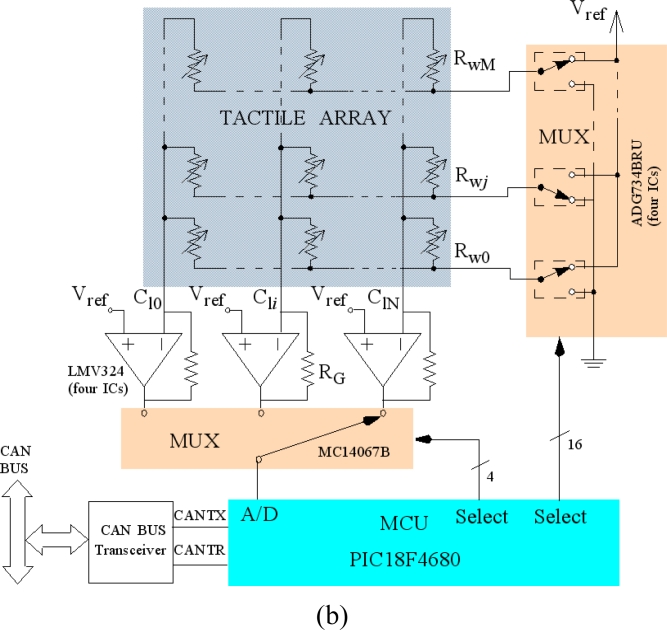
(**a**) Photograph of the electronics. (**b**) simplified schematics.

Figure 10(a) shows the local electronics of the smart tactile sensor. It is based on a microcontroller PIC18F4680 and is in charge of scanning the array, storing the data and sending it via CAN bus to a central processing unit. More capabilities can be added to the smart sensor by taking advantage of the microcontroller. Figure 10(b) shows a basic schematic of the main blocks in Figure 10(a) except the power supply module. The array is scanned and every tactel output is read.

The output voltage is given by the following expression:

$$V_{\text{out}}=(\frac{R_{G}}{R_{ij}}+1)V_{\text{ref}}$$

where *R_ij_* is the force dependant resistance of the element *ij* in the array, and *R_G_* is the resistance to set the gain of the transresistance amplifiers at the output of every column.

**Figure 11.**
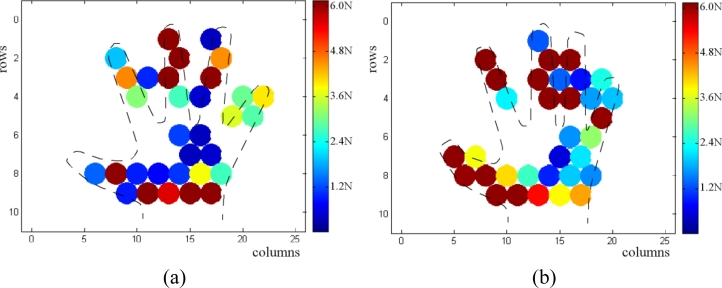
(**a**) and (**b**) two tactile images of a hand as registered by the tactile sensor.

Figure 11 shows the readings of the smart tactile sensor when its surface is pressed with the hand. The shape of the hand is clearly noticeable and the information about the existence of contact as well as the force exerted is provided.

**Figure 12.**
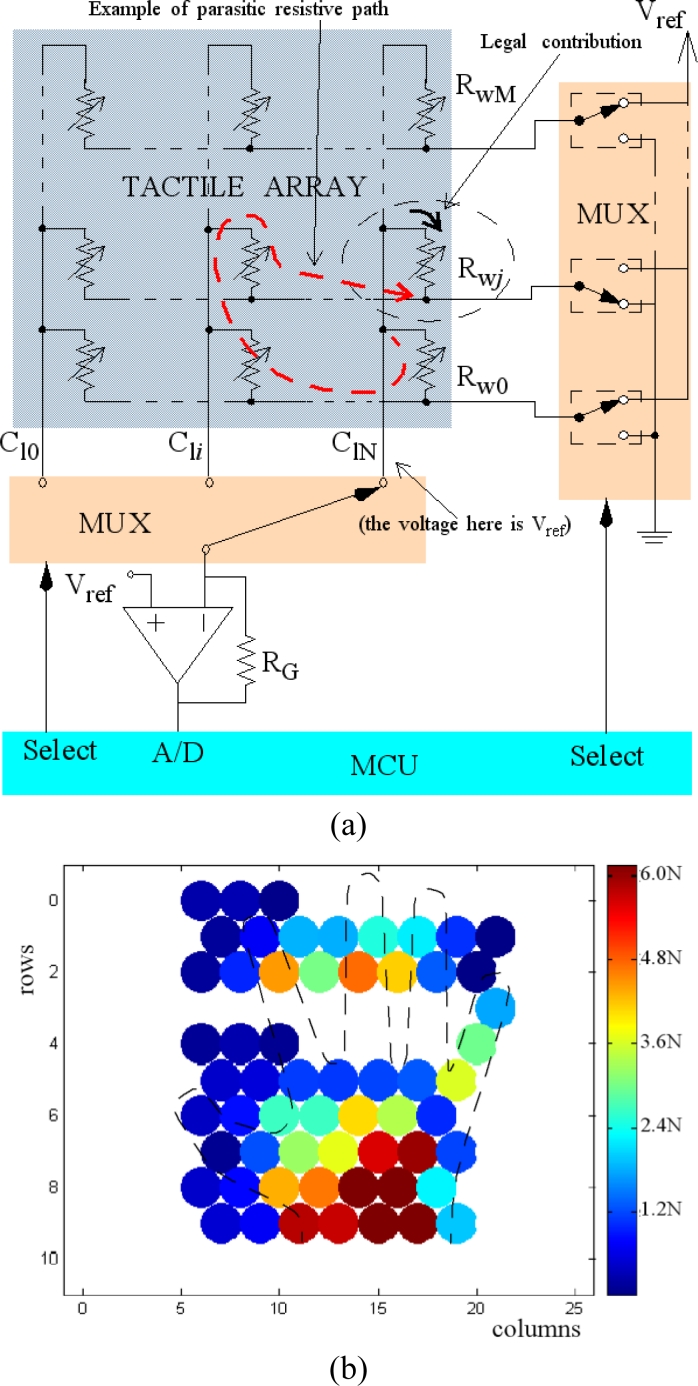
(**a**) Electronics that does not cancel crosstalk and (**b**) tactile image obatined from a hand on the sensor.

Note that one operational amplifier is required per column in Figure 10(b). Their purpose is to set the voltage *V_ref_* at the tracks of all columns. Since the voltage of all rows that do not contribute to the output is also set to *V_ref_*, any possible parasitic path is short circuited. This is a common grounding technique [31] in sensors developed from a continuous film of conductive rubber or polymer because parasitic resistors are present between tactels. We think it is worth highlighting here that these electronics must be used even when we have a discrete array. This is true in our case, because we have an array of force sensors. Nevertheless, once they are arranged in rows and columns and connected by the corresponding addressing tracks, multiple parasitic paths appear. One of them is depicted in Figure 12(a), where a simpler electronics layout that does not cancel the interferences is shown. The left part of Figure 12 shows the readings provided by this electronics. Note that crosstalk makes the shape of the hand indistinguishable and it is not possible to determine the orientation and size of the object on the sensor. Similarly, the implementation in [32] describes a technique where tactile sensors are formed by dispensing conductive polymer on copper electrodes. This is highlighted as a technique to reduce crosstalk, although grounding is also necessary.

Regarding the sensor bandwidth, reading out the whole array takes 12.8 ms, so it is much lower than the 100–200 ms we had set as a maximum. Moreover, distance between centers of tactels is 1.85 cm (see Figure 7(b)), so it is also lower that 3.8 cm which we had set as a goal taking into account the static spatial resolution of the human skin in the forearm. Finally, Table 3 shows data from the proposed sensor and from other reported tactile sensor patches oriented to cover large areas.

## 5. The Sensor in a Robot

**Figure 13.**
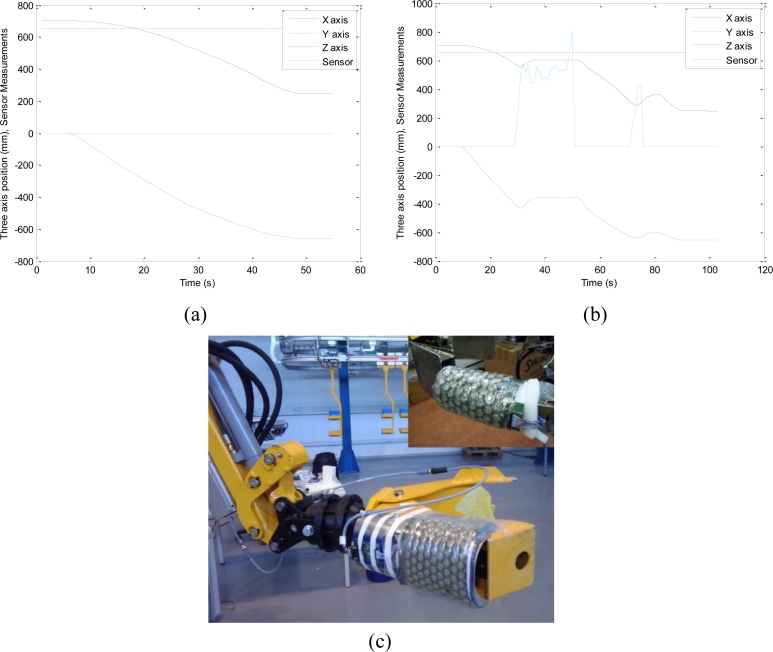
(**a**) Trajectories of the manipulator without contact, and (**b**) with two contacts. (**c**) Sensor mounted on the ALACRANE (the top right corner of the figure shows a detail where the sensor was mounted in a circular shape).

Figure 13(c) shows the sensor mounted on the arm of the rescue robot ALACRANE. This is a fully hydraulic robot that has been developed from a modified small demolition machine by Brokk^®^. The Main Arm has five DOF with five hydraulic cylinders. This redundant configuration increases its reachability of the end-effector. Its payload is 120 kg when it is fully extended, and 450 kg in the vicinity of the arm base.

An experiment has been carried out to show the performance of the sensor in this rescue robot. The robot has been programmed with the following reactive behavior. If contact is detected while it is doing some task, the manipulator moves back a predefined distance from the trajectory it was following. If there is no contact at this moment, the robot resumes the task. If there is still contact, the robot keeps still until the contact ceases.

Figure 13 illustrates this behavior. It shows the Cartesian coordinates *x*, *y*, *z*, referred to the base reference system of the robot as well as the average force registered by the tactels whose readings are higher than a certain threshold. Figure 13(a) shows a circular trajectory followed by the manipulator when no contact is detected, while Figure 13(b) shows the described reactive behavior when the manipulator makes contact with an obstacle twice. The first time the contact remains even when the manipulator has moved back, and the second time the contact is undone before the manipulator completes the backward movement, therefore the robot resumes its task. This behavior would keep people safe as well as objects surrounding the rescue robot and the robot itself.

**Figure 14.**
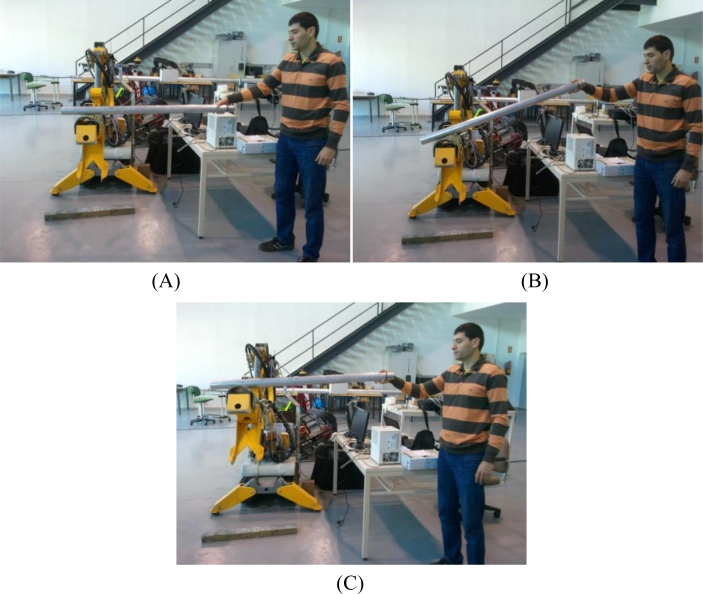
Photographs of the three main steps in the experiment where the robot cooperates with a human.

Another experiment was carried out to illustrate the applicability of the sensor. This time the robot takes the coordinates of the center of mass of the tactile image (*C_x_*, *C_y_*) to follow a certain trajectory. These coordinates are calculated by the microcontroller of the tactile patch as:

(1) $$C_{x}=\frac{\sum_{i=1}^{N}F_{i}x_{i}}{\sum_{i=1}^{N}F_{i}},\;\;C_{y}=\frac{\sum_{i=1}^{N}F_{i}y_{i}}{\sum_{i=1}^{N}F_{i}}$$

where (*x_i_*, *y_i_*) are the coordinates of the *i*-th tactel, *F_i_* is the force registered by it, and *N* is the number of tactels in the tactile sensor. Note that the obtained result takes into account the contact area and also the pressure distribution on this area. The trajectories of the center of mass when a dummy human is held in the arms of a robot are shown in [20] to illustrate the use of the sensor. Here we show the trajectory of the mass center and also how the robot can use this information in a cooperative task with a human. Figure 14 shows the starting situation (A), where a metal tube is held by the arm of the robot and by a human. Then the human lifts the end he is holding (B). This is perceived by the robot because the mass center is displaced.

**Figure 15.**
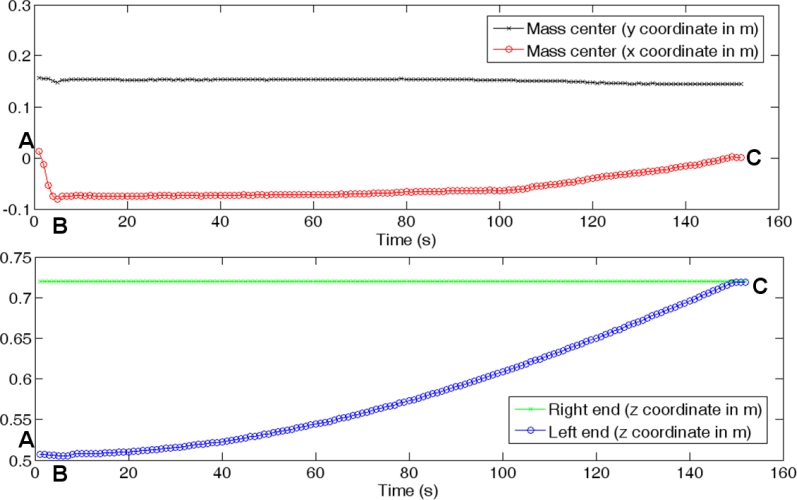
Trajectories of the mass center (top) and robot arm (curve in blue at the bottom graph).

This can be seen in Figure 15, where the trajectories of the center of mass and the robot arm are depicted. Now the robot lifts its arm until the location of the center of mass goes back to the initial value in (A). At this point, the heights of both ends of the tube are similar again (C), as can be seen in Figures 14 and 15.

## 6. Conclusions

A thorough description of the design of a large area tactile sensor has been presented. It is the first of this kind based on polymeric commercial force sensing resistors. This choice was made to achieve a robust design in a shorter time. Some modifications were needed to maintain the performance of the isolated force sensor, once it is incorporated to the tactile array on a flexible substrate. These modifications are explained as well as their impact on the response of the sensor. We can see the latter is negligible for good design practices that are discussed in the text. The performance of the sensor is shown through many experimental measurements of the tactel response as well as of the tactile sensor output. Finally, two examples illustrate its use in a rescue robot.

## Figures and Tables

**Table 1.** Interlink Electronics Standard 402 FSR Main Features.

**Size**	18.28 mm (diameter)
**Actuation Force**	0.1 N
**Durability**	10 Million actuations without failure
**Force Sensitive Range**	0.1–10 N 0.2% (single part)
**Force Repeatability**	0.6% (part to part)
**Hysteresis**	+10%
**Operating temp. range**	−30 °C to 70 °C

**Table 2.** Variations of Tactels.

**Tactel coordinates**	**Gain (Volts/N)**
(5,1)	0.57
(5,3)	0.63
(5,7)	0.65
(5,9)	0.59
(6,9)	0.62
(6,8)	0.68
(6,3)	0.55
(6,2)	0.65
(5,2)	0.55
(5,4)	0.65
(5,6)	0.69
(5,8)	0.61
(5,10)	0.55
(5,11)	0.57
(6,11)	0.57
(6,1)	0.47

Average	0.60
S.D.	0.06

**Table 3.** Performance data from the proposed and other reported tactile sensor patches.

**Author/Year**	**Transd. Method**	**Tech.**	**N° of Sensing Elements**	**Spatial Resol.**	**Sensor BW**	**Force/Press. Range**	**Force/Press. Sensitivity**
Ohmura [7]/2006	Optical	Flexible PCB	8 × 4	∼30 mm	5 kHz	500 kPa	
Shan [5]/2005	Piezores.	MEMS on Si, Flex.PCB	4 × 4	10 mm		2 N	228 mV/N (shear forces: 34 mV/N)
Heo [8]/2006	Optical		3 × 3	5 mm		5 N	1 mN
Cannata [10]/2008	Capacitive	Flexible PCB	12 (triangular patch)	2 tactels/cm	Up to 0.5 kHz		
Ulmen [11]/2010	Capacitive	Foam Layer PCB	4 × 4	15 mm	0.080 kHz	100 N	0.02 N
Shimojo [13]/2004	Conduct. Rubber		16 × 3	3 mm		12 N	0.2 MPa
Kerpa [14]/2003	Piezores.	PCB	10 × 23	15 mm	0.040 kHz	120 kPa	12 bits
Someya [20]/2004	FSR	Organic FET	16 × 16	2.54 mm	0.003 KHz	30 kPa	
Mukai [21]/2008	Silicon based FSR	Flexible PCB	8 × 8	18 mm	0.1 kHz	128 kPa	
Sthiel [23]/2006	FSR (QTC)	PCB	4 × 2				
Proposed	Polymer based FSR	Flexible PCB	16 × 9	18.5 mm	0.078 kHz	6 N	0.60 V/N
